# Supplementary material for: Mechanisms governing the pioneering and redistribution capabilities of the non-classical pioneer PU.1
Source: Nat Commun. 2020 Jan 21;11:402. doi: 10.1038/s41467-019-13960-2 (PMC6972792; doi:10.1038/s41467-019-13960-2)
Supplement: Supplementary file 7 — Source data [file 41467_2019_13960_MOESM7_ESM.zip › Source_Data/FigureS2/FigureS2B_MotifScanOutput/KmeansCluster12/homerResults.html]

/misc/data/analysis/project\_PU1/CTV1/motifs/basic/PU1\_Kmeans14.12/final - Homer de novo Motif Results


# Homer *de novo* Motif Results (/misc/data/analysis/project\_PU1/CTV1/motifs/basic/PU1\_Kmeans14.12/final)

Known Motif Enrichment Results  
Gene Ontology Enrichment Results  
If Homer is having trouble matching a motif to a known motif, try copy/pasting the matrix file into
STAMP  
More information on motif finding results: HOMER
| Description of Results
| Tips
  
Total target sequences = 1797  
Total background sequences = 46008  
\* - possible false positive  

|  |  |  |  |  |  |  |  |  |
| --- | --- | --- | --- | --- | --- | --- | --- | --- |
| Rank | Motif | P-value | log P-pvalue | % of Targets | % of Background | STD(Bg STD) | Best Match/Details | Motif File |
| 1 | C T G A C T G A T A C G T C G A A C T G A C T G C G T A C G T A T A C G G A C T A T C G | 1e-745 | -1.716e+03 | 75.96% | 13.33% | 38.1bp (69.7bp) | PU.1(ETS)/ThioMac-PU.1-ChIP-Seq(GSE21512)/Homer(0.957) More Information \| Similar Motifs Found | motif file (matrix) |
| 2 | G C A T C G T A T C G A A T G C G A T C C T G A G T A C G C T A | 1e-122 | -2.811e+02 | 34.67% | 12.50% | 52.8bp (65.5bp) | RUNX1(Runt)/Jurkat-RUNX1-ChIP-Seq(GSE29180)/Homer(0.971) More Information \| Similar Motifs Found | motif file (matrix) |
| 3 | C G A T A G C T A T G C G A T C A T C G A G C T G A C T A G C T G C A T | 1e-91 | -2.105e+02 | 30.38% | 11.88% | 50.7bp (70.9bp) | SpiB(ETS)/OCILY3-SPIB-ChIP-Seq(GSE56857)/Homer(0.777) More Information \| Similar Motifs Found | motif file (matrix) |
| 4 | A C T G A G T C A G C T A G C T A G T C A G T C C A G T A G T C G T A C G A T C A T G C G A T C | 1e-57 | -1.323e+02 | 21.59% | 8.79% | 53.7bp (70.1bp) | NCU02404(RRM)/Neurospora\_crassa-RNCMPT00238-PBM/HughesRNA(0.735) More Information \| Similar Motifs Found | motif file (matrix) |
| 5 | G C A T A C T G C T G A A C T G C G T A C G T A A C T G A C G T C T G A | 1e-43 | -9.961e+01 | 12.13% | 4.00% | 50.1bp (66.6bp) | RNP4F(RRM)/Drosophila\_melanogaster-RNCMPT00060-PBM/HughesRNA(0.725) More Information \| Similar Motifs Found | motif file (matrix) |
| 6 | A C T G C G A T A G C T A T G C A G T C G A C T A G T C G A C T A G C T A G C T C G A T A T C G | 1e-42 | -9.732e+01 | 6.29% | 1.16% | 55.2bp (70.1bp) | PI/MA0559.1/Jaspar(0.802) More Information \| Similar Motifs Found | motif file (matrix) |
| 7 | C T A G C T A G A T G C C A G T A T C G G C A T T C A G C T A G C T A G C A T G C T G A C T G A T A C G C A G T | 1e-41 | -9.542e+01 | 20.03% | 9.23% | 53.4bp (65.0bp) | ZNF467(Zf)/HEK293-ZNF467.GFP-ChIP-Seq(GSE58341)/Homer(0.754) More Information \| Similar Motifs Found | motif file (matrix) |
| 8 | A G T C G A C T A C G T A G T C A G T C A C G T A C G T A T G C T G A C G C A T T C A G | 1e-41 | -9.482e+01 | 19.37% | 8.79% | 57.4bp (69.3bp) | RBM5(Znf)/Homo\_sapiens-RNCMPT00055-PBM/HughesRNA(0.738) More Information \| Similar Motifs Found | motif file (matrix) |
| 9 | T C G A A C G T A C T G C G T A T A G C C G A T G T A C G T C A A G C T G T A C | 1e-41 | -9.444e+01 | 7.96% | 1.93% | 51.9bp (60.8bp) | Fra1(bZIP)/BT549-Fra1-ChIP-Seq(GSE46166)/Homer(0.989) More Information \| Similar Motifs Found | motif file (matrix) |
| 10 | G T C A T C G A C T A G A C T G G T C A C T G A C A T G G A C T C A T G C T A G A G T C C G A T | 1e-35 | -8.128e+01 | 5.90% | 1.23% | 56.5bp (65.8bp) | ETS:RUNX(ETS,Runt)/Jurkat-RUNX1-ChIP-Seq(GSE17954)/Homer(0.763) More Information \| Similar Motifs Found | motif file (matrix) |
| 11 | T A G C C T G A C G A T C G T A C A T G C T A G T A C G C T A G C G T A G C T A A T G C A G C T T A G C G T C A | 1e-33 | -7.698e+01 | 3.56% | 0.42% | 44.2bp (62.1bp) | RO3G\_00049(RRM)/Rhizopus\_oryzae-RNCMPT00205-PBM/HughesRNA(0.732) More Information \| Similar Motifs Found | motif file (matrix) |
| 12 | G A C T A C T G C A G T T C A G A C T G C T A G G T C A C T A G C A T G | 1e-30 | -6.979e+01 | 30.33% | 18.75% | 58.0bp (65.2bp) | Egr2(Zf)/Thymocytes-Egr2-ChIP-Seq(GSE34254)/Homer(0.804) More Information \| Similar Motifs Found | motif file (matrix) |
| 13 | A T C G T G A C A T C G C A T G C G T A A G C T T A C G G A C T T A C G | 1e-28 | -6.673e+01 | 4.62% | 0.92% | 47.4bp (58.5bp) | SPDEF/MA0686.1/Jaspar(0.768) More Information \| Similar Motifs Found | motif file (matrix) |
| 14 | G A T C C G T A A T C G G A C T G C A T A G T C G T A C C G A T A G T C A T G C G A T C G T A C | 1e-27 | -6.363e+01 | 8.96% | 3.24% | 52.6bp (63.7bp) | HNRNPH2(RRM)/Homo\_sapiens-RNCMPT00160-PBM/HughesRNA(0.759) More Information \| Similar Motifs Found | motif file (matrix) |
| 15 | G T C A G C T A T C G A T A G C G T A C G C T A G T A C G C A T A C G T A G T C G T C A G T C A C T G A | 1e-26 | -6.191e+01 | 3.23% | 0.46% | 57.3bp (66.8bp) | PH0113.1\_Nkx2-4/Jaspar(0.767) More Information \| Similar Motifs Found | motif file (matrix) |
| 16 | G T A C G C T A C G T A C G T A C T G A A C T G A T C G T C G A G C T A A T C G A C T G | 1e-26 | -6.069e+01 | 5.45% | 1.40% | 46.5bp (69.1bp) | SPIC/MA0687.1/Jaspar(0.711) More Information \| Similar Motifs Found | motif file (matrix) |
| 17 | T C A G T C G A T A C G T A G C T A C G T C A G C T G A T C G A T C G A A T G C G T A C T G C A | 1e-24 | -5.623e+01 | 4.56% | 1.07% | 54.6bp (66.7bp) | che-1/MA0260.1/Jaspar(0.703) More Information \| Similar Motifs Found | motif file (matrix) |
| 18 | A T G C T C A G T A C G C A G T A G C T C G T A T A G C A T G C A C T G | 1e-24 | -5.591e+01 | 23.59% | 14.26% | 57.6bp (67.1bp) | MYB98(MYB)/col-MYB98-DAP-Seq(GSE60143)/Homer(0.796) More Information \| Similar Motifs Found | motif file (matrix) |
| 19 | A T G C C G T A A T C G C T G A C T A G C T A G G T C A C T G A A C T G T A G C A G T C G C A T | 1e-24 | -5.562e+01 | 4.67% | 1.14% | 60.8bp (61.7bp) | GCR1/Literature(Harbison)/Yeast(0.871) More Information \| Similar Motifs Found | motif file (matrix) |
| 20 | G C T A C T A G C T A G G A T C C T G A A T C G G C A T C G A T C G T A A G T C A C G T | 1e-22 | -5.144e+01 | 9.96% | 4.33% | 54.7bp (65.4bp) | PB0149.1\_Myb\_2/Jaspar(0.851) More Information \| Similar Motifs Found | motif file (matrix) |
| 21 | G A T C G A T C G T C A T A G C A G C T A G C T A G T C C G A T C A T G A G T C G A C T C A G T A C G T | 1e-21 | -5.018e+01 | 3.45% | 0.69% | 46.4bp (70.3bp) | SpiB(ETS)/OCILY3-SPIB-ChIP-Seq(GSE56857)/Homer(0.775) More Information \| Similar Motifs Found | motif file (matrix) |
| 22 | A G T C A G T C T G C A A G T C A C G T A G C T A G T C G T A C T A G C A G T C C T G A | 1e-21 | -5.015e+01 | 7.12% | 2.59% | 57.4bp (68.5bp) | Zfp281(Zf)/ES-Zfp281-ChIP-Seq(GSE81042)/Homer(0.760) More Information \| Similar Motifs Found | motif file (matrix) |
| 23 | C G T A T G C A A G T C A G T C T C G A G A T C T G C A C T G A A C G T A G C T G A C T A T G C T A G C C A G T | 1e-21 | -4.957e+01 | 1.45% | 0.07% | 52.0bp (45.8bp) | GFY(?)/Promoter/Homer(0.697) More Information \| Similar Motifs Found | motif file (matrix) |
| 24 | A T C G C G A T A G C T A T G C A G C T A C G T G T A C G T A C C A G T A G T C | 1e-20 | -4.834e+01 | 6.18% | 2.10% | 53.3bp (74.3bp) | TRA2(RRM)/Drosophila\_melanogaster-RNCMPT00078-PBM/HughesRNA(0.728) More Information \| Similar Motifs Found | motif file (matrix) |
| 25 | G T A C G T A C G A T C A G T C T G A C A G T C A G T C A G T C C G T A G T A C G A T C | 1e-20 | -4.798e+01 | 18.48% | 10.75% | 56.9bp (64.9bp) | ZNF740/MA0753.1/Jaspar(0.858) More Information \| Similar Motifs Found | motif file (matrix) |
| 26 | A T C G C T G A A C T G G C A T A C G T A G T C A G T C G A C T A T C G G A C T C T G A C A G T | 1e-19 | -4.584e+01 | 3.28% | 0.69% | 45.9bp (62.6bp) | EWS:ERG-fusion(ETS)/CADO\_ES1-EWS:ERG-ChIP-Seq(SRA014231)/Homer(0.739) More Information \| Similar Motifs Found | motif file (matrix) |
| 27 | A T C G G A T C C G A T C A T G T A G C C G A T A T C G G T C A T A C G G C A T G A C T | 1e-19 | -4.582e+01 | 9.18% | 4.06% | 56.9bp (65.0bp) | MafA(bZIP)/Islet-MafA-ChIP-Seq(GSE30298)/Homer(0.738) More Information \| Similar Motifs Found | motif file (matrix) |
| 28 | C A G T T G C A A T C G A T G C C T A G C T A G C G T A A G C T T C A G A C G T C T A G A C T G | 1e-19 | -4.569e+01 | 3.01% | 0.57% | 55.3bp (66.6bp) | ETS:RUNX(ETS,Runt)/Jurkat-RUNX1-ChIP-Seq(GSE17954)/Homer(0.792) More Information \| Similar Motifs Found | motif file (matrix) |
| 29 | G A C T C T G A C T G A G A T C T C A G A C T G G A C T A C G T | 1e-18 | -4.308e+01 | 10.68% | 5.22% | 61.2bp (67.8bp) | PB0046.1\_Mybl1\_1/Jaspar(0.895) More Information \| Similar Motifs Found | motif file (matrix) |
| 30 | A C G T A T G C A C G T A C G T C G T A A C G T A G T C C A T G | 1e-18 | -4.281e+01 | 7.23% | 2.92% | 54.6bp (67.2bp) | DAL80/MA0289.1/Jaspar(0.950) More Information \| Similar Motifs Found | motif file (matrix) |
| 31 | G T A C G A T C C G T A A T G C G A C T C G T A A C T G C T G A A C T G A C T G T A C G T G A C C T A G | 1e-17 | -4.127e+01 | 1.56% | 0.13% | 68.3bp (54.9bp) | BORIS(Zf)/K562-CTCFL-ChIP-Seq(GSE32465)/Homer(0.873) More Information \| Similar Motifs Found | motif file (matrix) |
| 32 | A T C G G T A C A G C T A C T G A C G T A G C T C T A G G T C A | 1e-17 | -4.108e+01 | 35.00% | 25.51% | 56.7bp (66.6bp) | POL010.1\_DCE\_S\_III/Jaspar(0.736) More Information \| Similar Motifs Found | motif file (matrix) |
| 33 | G A C T T C G A C A T G G A T C C G T A C T A G G A T C G C T A A T C G G T A C | 1e-17 | -3.936e+01 | 17.86% | 10.92% | 58.5bp (67.0bp) | PRDM9(Zf)/Testis-DMC1-ChIP-Seq(GSE35498)/Homer(0.741) More Information \| Similar Motifs Found | motif file (matrix) |
| 34 | T A C G C G A T A C T G A T C G A G C T G C A T A C T G A C T G C A G T A C T G A T C G G A C T C G A T C A G T | 1e-16 | -3.787e+01 | 4.73% | 1.59% | 51.8bp (58.9bp) | AT1G24250(Orphan)/col-AT1G24250-DAP-Seq(GSE60143)/Homer(0.762) More Information \| Similar Motifs Found | motif file (matrix) |
| 35 | G A C T C A G T G T A C G C A T A C G T A G T C C G T A T G C A C T G A A T C G | 1e-16 | -3.743e+01 | 11.46% | 6.11% | 56.9bp (67.6bp) | pan/dmmpmm(Papatsenko)/fly(0.818) More Information \| Similar Motifs Found | motif file (matrix) |
| 36 | C G T A C T G A T C G A C T G A C T A G A C T G C G T A G C T A G T A C G C A T G A T C G T C A A C G T | 1e-16 | -3.719e+01 | 1.67% | 0.20% | 41.8bp (70.3bp) | RME1(MacIsaac)/Yeast(0.693) More Information \| Similar Motifs Found | motif file (matrix) |
| 37 | C T G A A T C G C G T A C A T G C A T G G T C A G T C A G T A C G C A T A T C G T G A C | 1e-16 | -3.713e+01 | 5.34% | 1.97% | 53.9bp (69.1bp) | PU.1(ETS)/ThioMac-PU.1-ChIP-Seq(GSE21512)/Homer(0.741) More Information \| Similar Motifs Found | motif file (matrix) |
| 38 | T A C G C A G T A C T G C A T G A T C G C A G T T A C G C A T G C T A G T C A G C G T A C T A G T C A G | 1e-15 | -3.670e+01 | 7.35% | 3.24% | 57.8bp (62.1bp) | KLF5/MA0599.1/Jaspar(0.733) More Information \| Similar Motifs Found | motif file (matrix) |
| 39 | A C G T A C G T A C G T T A G C T G A C G C A T A C T G | 1e-15 | -3.645e+01 | 43.29% | 33.79% | 58.3bp (67.0bp) | EWS:ERG-fusion(ETS)/CADO\_ES1-EWS:ERG-ChIP-Seq(SRA014231)/Homer(0.872) More Information \| Similar Motifs Found | motif file (matrix) |
| 40 | C A T G C G T A C A T G T A C G G T C A C G T A T A C G C G A T C T A G A T C G C G T A A C T G C T G A | 1e-15 | -3.596e+01 | 4.90% | 1.74% | 54.1bp (69.6bp) | SRSF2(RRM)/Homo\_sapiens-RNCMPT00072-PBM/HughesRNA(0.690) More Information \| Similar Motifs Found | motif file (matrix) |
| 41 | G C A T A G C T A T G C A G T C A C G T A T G C G A C T A G C T G A T C G A T C G C A T T A G C A C G T | 1e-15 | -3.578e+01 | 3.01% | 0.74% | 50.4bp (61.0bp) | Trl/dmmpmm(SeSiMCMC)/fly(0.724) More Information \| Similar Motifs Found | motif file (matrix) |
| 42 | A G T C T C G A T A G C A G T C G T C A A T C G T A C G A C G T A C T G A C T G G A T C C T A G T A G C | 1e-14 | -3.379e+01 | 1.22% | 0.10% | 43.5bp (69.3bp) | CTCF/MA0531.1/Jaspar(0.820) More Information \| Similar Motifs Found | motif file (matrix) |
| 43 | C G T A A G T C C G A T T C G A A T G C C T G A G T C A A G C T C A G T A G T C A G T C A T G C G T C A A C T G | 1e-14 | -3.286e+01 | 1.34% | 0.13% | 49.8bp (65.2bp) | GFY(?)/Promoter/Homer(0.897) More Information \| Similar Motifs Found | motif file (matrix) |
| 44 | T A G C G T C A C G T A T G C A A G T C G T A C T G C A G T A C T A G C G C T A T A C G G T A C | 1e-14 | -3.265e+01 | 2.23% | 0.45% | 52.3bp (66.9bp) | AT1G24250(Orphan)/col-AT1G24250-DAP-Seq(GSE60143)/Homer(0.696) More Information \| Similar Motifs Found | motif file (matrix) |
| 45 | T A C G A G T C A G C T A G C T A G T C G T C A C T A G A G T C C G A T T G A C C G A T C T A G A G T C | 1e-13 | -3.160e+01 | 0.83% | 0.03% | 36.4bp (69.2bp) | GATA12(C2C2gata)/col-GATA12-DAP-Seq(GSE60143)/Homer(0.591) More Information \| Similar Motifs Found | motif file (matrix) |
| 46 | C G T A T C A G A C G T C A T G C T G A A G C T T C G A G A C T G A T C C T G A | 1e-13 | -3.145e+01 | 1.34% | 0.14% | 55.3bp (62.1bp) | Optix/dmmpmm(Noyes\_hd)/fly(0.815) More Information \| Similar Motifs Found | motif file (matrix) |
| 47 | A C G T A T C G C T G A A T C G A C G T A G T C G T C A A T G C T G A C C G T A C T A G A G C T T A G C | 1e-13 | -3.095e+01 | 0.83% | 0.03% | 49.0bp (56.7bp) | FOSL1/MA0477.1/Jaspar(0.738) More Information \| Similar Motifs Found | motif file (matrix) |
| 48 | T A C G T G A C C G T A C A T G G A T C G A C T C A T G A G C T C G A T | 1e-13 | -3.077e+01 | 12.02% | 6.98% | 56.5bp (69.4bp) | Ascl2/MA0816.1/Jaspar(0.903) More Information \| Similar Motifs Found | motif file (matrix) |
| 49 | C G T A C T G A T A C G T C G A A C G T T C A G T G C A A G C T C A T G G C A T G A T C | 1e-13 | -3.061e+01 | 3.01% | 0.85% | 50.5bp (58.1bp) | Atf3/MA0605.1/Jaspar(0.769) More Information \| Similar Motifs Found | motif file (matrix) |
| 50 | C T A G C A G T C G A T C G T A C G T A C G T A C A T G C T G A C A G T C G T A | 1e-12 | -2.991e+01 | 2.45% | 0.59% | 56.2bp (66.2bp) | Ct/dmmpmm(Noyes\_hd)/fly(0.707) More Information \| Similar Motifs Found | motif file (matrix) |
| 51 | G T C A T C G A A G T C T A G C C A T G G T A C C G T A A C T G A G T C G A T C | 1e-12 | -2.947e+01 | 3.06% | 0.91% | 54.7bp (64.1bp) | Bgb::run/MA0242.1/Jaspar(0.815) More Information \| Similar Motifs Found | motif file (matrix) |
| 52 | A C G T G C A T C G A T G C T A C G A T G T A C C G T A T A G C | 1e-12 | -2.851e+01 | 19.76% | 13.50% | 58.5bp (65.4bp) | Optix/dmmpmm(Noyes\_hd)/fly(0.853) More Information \| Similar Motifs Found | motif file (matrix) |
